# Implementation of a Language-Concordant, Culturally Tailored Inpatient Lactation Program

**DOI:** 10.1001/jamanetworkopen.2025.0274

**Published:** 2025-03-07

**Authors:** Nikita S. Kalluri, Elena Padilla-Garza, Tessa Kehoe, Chloe Andrews, Julianna Schantz-Dunn, Jennifer Riley, Mary Pomerleau, Anne CC Lee, Sarbattama Sen

**Affiliations:** 1 Department of Pediatrics, UMass Chan Medical School, Worcester, Massachusetts; 2 Department of Pediatric Newborn Medicine, Brigham and Women’s Hospital, Boston, Massachusetts; 3 Harvard Medical School, Boston, Massachusetts; 4 Women and Infant’s Hospital, Providence, Rhode Island; 5 Department of Obstetrics and Gynecology, Brigham and Women’s Hospital, Boston, Massachusetts; 6 Department of Pediatrics, Warren Alpert Medical School of Brown University, Providence, Rhode Island

## Abstract

**Importance:**

Benefits of breastfeeding are extensive, but racial and ethnic disparities persist. Mama Sana (Spanish for *healthy mother*) is a bilingual, culturally tailored program that aims to reduce breastfeeding inequities.

**Objective:**

To examine differences in lactation support and breastfeeding outcomes among Spanish-speaking Hispanic birthing parents who participated in Mama Sana compared with a historical control (pre–Mama Sana) group.

**Design, Setting, and Participants:**

In this cohort study, patients who participated in the Mama Sana program during their delivery hospitalization from January 2022 to September 2023 were compared with a historical control group who delivered prior to the implementation of the program (July 2019 to December 2021). The study took place at an academic medical center in Boston with a high-risk obstetric service. Data were analyzed from January to September 2024.

**Exposure:**

Beginning in January 2022, a bilingual native Spanish-speaker licensed as a registered dietitian and certified lactation counselor provided lactation support to participants during their delivery hospitalization with follow-up phone calls after discharge (until 6 months post partum).

**Main Outcomes and Measures:**

The primary outcome was exclusive breastfeeding at discharge. Secondary outcomes included any breastfeeding at discharge, any breastfeeding and exclusive breastfeeding at 6 weeks post partum, and change in maternal body mass index (BMI). Process measures included inpatient lactation consult placement and receipt of any and language-concordant lactation support. The χ^2^ test was used to analyze outcomes, process measures, and balancing measures.

**Results:**

In this study, 417 patients were included (Mama Sana group, 175 patients; mean [SD] age, 28.9 [6.1] years; pre–Mama Sana group: 242 patients; mean [SD] age, 29.8 [6.1] years). Sociodemographic characteristics including age and BMI were similar between groups. Mama Sana participants were less likely to be multiparous or to have diabetes compared with the pre–Mama Sana group. At hospital discharge, 36 of 175 Mama Sana participants (20.6%) were exclusively breastfeeding compared with 39 of 242 pre–Mama Sana (16.1%) (adjusted risk difference [aRD], 2.5%; 95% CI, −1.2 to 6.4). Significantly more Mama Sana participants engaged in any breastfeeding prior to hospital discharge (172 of 175 [98.3%] vs 222 of 242 [91.7%]; aRD, 7.1%; 95% CI, 2.8% to 11.5%) and at the 6 week postpartum visit (81 of 102 [79.4%] vs 109 of 170 [64.1%]; aRD, 15.6%; 95% CI, 4.8% to 26.4%) compared with the pre–Mama Sana group. More Mama Sana participants had a lactation consult placed (109 of 175 [62.3%] vs 108 of 242 [44.6%]; *P* < .001) and received lactation support in Spanish (175 of 175 [100%] vs 49 of 242 [20.3%]; *P* < .001) than those in the pre–Mama Sana group.

**Conclusions and Relevance:**

In this cohort study, Mama Sana’s language-concordant, culturally tailored lactation program was associated with higher rates of lactation support and some breastfeeding outcomes, which suggests the program may be a useful approach to perinatal care equity.

## Key Points

**Question:**

Is implementation of a language-concordant and culturally tailored lactation program associated with increased breastfeeding rates?

**Findings:**

In this cohort study, participation in the Mama Sana program was not associated with a statistically significant increase in exclusive breastfeeding at discharge vs historical controls, the study's primary outcome. Mama Sana participants experienced more inpatient lactation support and counseling and had significantly higher rates of any breastfeeding at hospital discharge and at 6 weeks post partum.

**Meaning:**

These findings suggest that providing a language-concordant and culturally tailored lactation support program may improve breastfeeding, providing a pathway to equitable perinatal care provision for Spanish-speaking Hispanic patients.

## Teaser

This cohort study examines breastfeeding outcomes among mothers who participated in a Spanish-language breastfeeding program.

## Introduction

Dose-dependent health benefits of breastfeeding for newborns and mothers have been extensively described.^1,2,3,4^ Longer breastfeeding duration and intensity is associated with lower postpartum weight retention and lower risk of type 2 diabetes and breast and ovarian cancers.^5,6,7^ Breastfed infants have lower risk of all-cause infant mortality,^8^ infections,^3,9^ obesity,^4^ and improved child development.^10^ Given these benefits, the American College of Obstetrics and Gynecology and American Academy of Pediatrics recommend exclusive breastfeeding (EBF) for at least the first 6 months to support health outcomes.^2,11^ However, in the US, there are stark drop-offs in breastfeeding rates through 6 months of life, with notable disparities based on race, ethnicity, and language. In 2019, 87% of Hispanic birthing parents initiated breastfeeding, but only 24% were exclusively breastfeeding 6 months post partum.^12,13^ This is well below the Healthy People 2030 goal of 42%, and lower than the non-Hispanic White population, of whom 29% exclusively breastfeed 6 months post partum.^14^ The Agency for Healthcare Research and Quality has identified culturally and linguistically appropriate services (CLAS) as a focus area to target health disparities.^15^

Limited CLAS lactation support is identified as a barrier to Spanish-speaking Hispanic patients achieving their breastfeeding goals.^16,17^ Brigham and Women’s Hospital (BWH) data from 2019 to 2020 demonstrated disparities based on race, ethnicity, and preferred language, with 54% of NHW infants exclusively breastfeeding at hospital discharge compared with 14.5% of Hispanic infants. Only 4.7% of Spanish-speaking patients on public insurance were exclusively breastfeeding at discharge. Patients identified lack of lactation support in their native language as a barrier, with only 2% receiving lactation support from a Spanish speaker and 18% receiving lactation support with documented interpretation. Timely interpreter access is challenging, and it can be difficult to build rapport using phone or tablet-based interpretation.^18^ In response, the Mama Sana (ie, Healthy Mother) program was developed to provide language-concordant, culturally tailored breastfeeding counseling to Spanish-speaking Hispanic patients at BWH.

The objectives of this study were to (1) compare access with lactation support between Mama Sana participants at BWH and a pre–Mama Sana historical control group and (2) determine whether Mama Sana participation was associated with improved breastfeeding rates at hospital discharge and at 6 weeks post partum.

## Methods

### Design and Population

This cohort study followed the Strengthening the Reporting of Observational Studies in Epidemiology (STROBE) reporting guideline.^19^ This project was approved as exempt by the institutional review board at BWH because deidentified data was collected for clinical improvement. Consent was waived for the historical control. Participants in the Mama Sana program provided electronic consent using a secure web-based form.

This was a pre-post study at BWH, an academic, tertiary care center with a high-risk obstetric service in Boston, Massachusetts. We included patients who delivered a term infant admitted to the BWH Well Newborn Nursery between July 2019 and September 2023. We excluded dyads if the infant was admitted to the neonatal intensive care unit (NICU) or received formula for medical indications (low blood glucose, hyperbilirubinemia, or significant weight loss). 

All data were collected via manual abstraction from the hospital electronic medical record (EMR). To ensure high-quality, reproducible abstraction, 2 authors (N.S.K. and E.P.G.) performed an EMR audit for study outcomes (feeding type at hospital discharge and 6-week visit, and postpartum BMI) on 60 randomly identified records, with discrepancy in less than 5% of records, which were resolved by team consensus.

### Mama Sana Program

Program staff screened for Spanish-speaking Hispanic postpartum patients daily using the EMR and reviewed eligibility with the clinical team. Patients could also be referred to Mama Sana by clinical staff if patients expressed interest or if staff felt they would benefit. One native Spanish-speaker of Hispanic ethnicity licensed as a registered dietitian (RD) and certified lactation counselor (CLC) provided counseling to participants at 2 time points:

1. Delivery hospitalization
  - Initial Mama Sana RD/CLC meeting to provide hands-on lactation support
  - Daily RD/CLC visits to provide hands-on lactation support, as needed, throughout the hospitalization
  - Joint consultation with Mama Sana RD/CLC and International Board Certified Lactation Consult (IBCLC) clinician if needed
2. Post partum
  - Lactation support warm line, with response within 24 hours from Spanish-speaking RD/CLC, available 5 days a week
  - Follow-up phone calls (7 to 14 days, 3 months, 6 months) to address breastfeeding concerns and connect to community resources as needed (eg, Stronger Generations, Vital Village, Postpartum Support International)^20,21,22^

To ensure cultural humility, the program RD/CLC honed her practice based on focus group feedback and collaboration with peer counselors. Counseling was tailored to incorporate common terminology in the most represented countries of origin at BWH. This allowed her to address a broad range of lactation practices, and motivational interviewing expertise and personal cultural experience being from Latin America facilitated respectful identification of additional practices.

### Pre–Mama Sana Standard of Care

Postpartum lactation support was provided by the English-speaking BWH lactation team (CLCs and IBCLCs). Telephone or video interpretation could be used at clinician discretion. Both before and after program implementation, bedside nurses also provided hands-on lactation support based on their experience and comfort. BWH has been designated a Baby-Friendly hospital by Baby-Friendly USA, an organization that supports best practices in infant feeding care, and therefore provides antenatal counseling regarding the importance of breastfeeding and care practices to facilitate breastfeeding.^23^

### Outcomes

Research staff abstracted in-hospital breastfeeding measures from nursing flowsheet documentation in the EMR, and postpartum visit measures (including breastfeeding data and BMI) from physician documentation in the postpartum visit note in the EMR. During the hospitalization, if there was no EMR documentation of formula receipt, infants were defined as exclusively breastfeeding, evaluated as a binary variable. Infants who received donor milk were considered to be exclusively breastfeeding if no formula was documented, in line with Baby-Friendly Hospital Initiative definitions.^23^ We defined any breastfeeding as documented breastfeeding or administration of pumped mother’s milk, and formula, defined as a binary variable. At the postpartum visit, physicians documented breastfeeding and/or formula. If there was no mention or either feeding type, postpartum feeding type was categorized as missing. Change in maternal BMI was defined as the change between 6-week postpartum BMI and prepregnancy BMI, evaluated as a continuous variable. Our primary outcome was exclusive breastfeeding at hospital discharge. Secondary outcomes included any breastfeeding at discharge, exclusive breastfeeding at 6 weeks post partum, any breastfeeding at 6 weeks post partum, and change in BMI.

Process measures included the placement of a lactation consult in the EMR and the receipt of lactation support. We categorized receipt of lactation support as any support or Spanish-language support (counseling with a documented interpreter or counselor with Spanish primary language). A subset of Spanish-language support was language-concordant support (counselor with Spanish primary language). In both periods, this was assessed from EMR documentation. We included balancing measures (defined as unintended consequences caused by a process change)^24^ that could be associated with inadequate breastfeeding, including infant hyperbilirubinemia (as documented in the physician medical record), hypoglycemia requiring treatment (dextrose gel or dextrose-containing intravenous fluids), and percentage of infant weight loss at discharge.

We collected additional covariates previously identified in the literature to impact breastfeeding, including maternal age, prepregnancy maternal BMI, multiparity, mode of delivery, breastfeeding within 1 hour of delivery, and maternal medical conditions.^7,16,25,26,27^ Due to limitations in chart abstraction, we were unable to further assess sociodemographic characteristics or health-related social needs, such as income, parental education, or resource difficulties. Our goal sample size of 200 per group was based on a risk difference of 7%, which approximates the observed difference in trials of breastfeeding promotion, with 80% power and α = .05.

### Analytic Approach

We compared dyad characteristics, outcomes, process measures, and balancing measures between Mama Sana participants and the historical control (pre–Mama Sana). As a secondary analysis, we compared outcomes and process and balancing measures between all dyads with infants born after Mama Sana implementation (epoch 2) to the historical control group to account for potential referral bias in breastfeeding outcomes (eTables 1 to 3 in Supplement 1).

### Statistical Analysis

First, we compared demographic characteristics between Mama Sana participants and the historical control using χ^2^ analyses for categorical variables and *t* tests for continuous variables. We compared outcomes, process measures, and balancing measures using the χ^2^ test. We developed modified Poisson regression models for dichotomous outcomes to generate relative risk ratios (RRs) and risk differences (RDs) with their 95% CIs to analyze associations between Mama Sana participation and outcomes of interest, with the historical control as reference.^28,29,30,31,32^ We adjusted for potential confounders, including parity, mode of delivery, and breastfeeding within 1 hour of life. Statistical analyses were performed using SAS OnDemand for Academics (SAS Institute).^31^ We used *P* < .05 as our threshold for significance, and all tests were 2-sided. Data were analyzed from January to September 2024.

## Results

**Figure 1.**
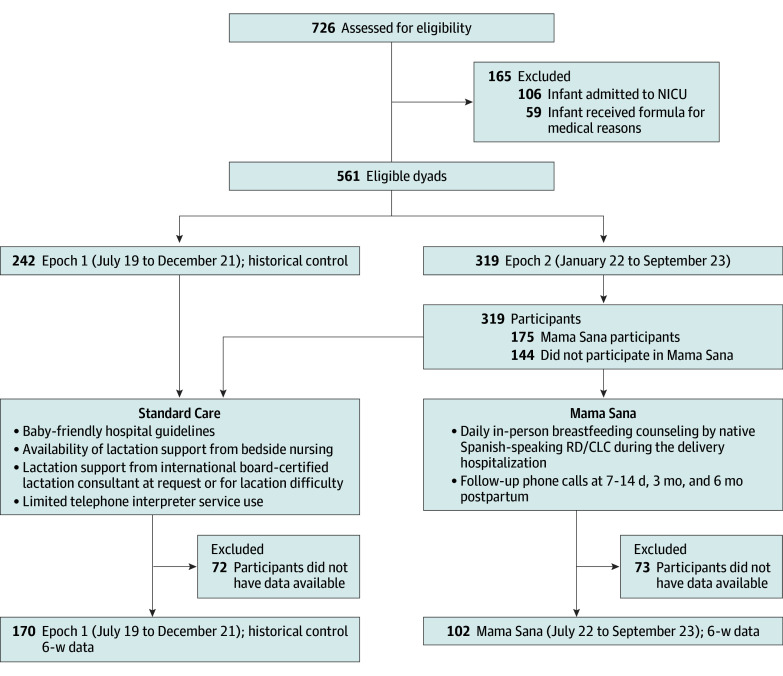
Spanish-Speaking Hispanic Patients Evaluated for Inclusion NICU indicates neonatal infant care unit; RD/CLC, registered dietitian and certified lactation counselor.

We assessed 726 dyads for eligibility from July 2019 to September 2023, with 561 eligible dyads and 417 included in the analysis. We excluded 106 dyads because of infant NICU admission and 59 dyads because the infant received formula for medical indications. Of remaining dyads, 43% delivered in the historical cohort (epoch 1: 242 dyads from July 2019 to December 2021) and 57% delivered after Mama Sana implementation (epoch 2: 319 dyads from January 2022 to September 2023). Of those in epoch 2, 55% participated in Mama Sana (175 of 319 dyads), based upon program availability and staffing (Figure 1). For 6-week postpartum breastfeeding outcomes, 102 of 175 Mama Sana participants (58%) and 170 of 242 of the historical control (70%) had data available.

Of the 417 participants included in study, the mean (SD) age of participants in the Mama Sana group was 28.9 (6.1) years and 29.8 (6.1) years in the pre–Mama Sana group. Baseline characteristics of participants and the historical control are shown in Table 1. Fewer Mama Sana participants were multiparous, had diabetes, breastfed within 1 hour, and performed documented skin-to-skin after delivery compared with the historical control. A comparison of baseline characteristics between Mama Sana participants and nonparticipants who delivered within epoch 2 can be found in eTable 1 in Supplement 1.

**Table 1.** Baseline Characteristics of Spanish-Speaking Hispanic Dyads by Group

Characteristics	Participants, No. (%)		*P* value
	Pre–Mama Sana, n = 242^a^	Mama Sana participants, n = 175	
Maternal characteristics			
Age, mean (SD), y	29.8 (6.1)	28.9 (6.1)	.15
Multiparous	166 (68.6)	98 (56.0)	.008
Cesarean section delivery	75 (31.0)	63 (36.0)	.28
Diabetes during pregnancy	32 (13.2)	11 (6.3)	.02
Maternal hypertension	15 (6.2)	9 (5.1)	.65
Preeclampsia	5 (2.1)	3 (1.7)	.80
Prepregnancy BMI, mean (SD)^b^	28.6 (6.2)	27.6 (5.6)	.18
Delivery BMI, mean (SD)^c^	32.8 (4.7)	31.9 (5.8)	.11
Skin-to-skin after delivery	186 of 240 (77.5)	117 of 171 (68.4)	.04
BF within 1 h	139 of 241 (57.7)	83 of 174 (47.7)	.04
Infant characteristics			
GA at delivery, mean (SD), wk	39.3 (1.1)	39.4 (1.2)	.22
Birth weight, mean (SD), kg	3.4 (0.4)	3.4 (0.4)	.67
Sex			
Male	119 (49.2)	97 (55.4)	.21
Female	123 (50.8)	78 (44.6)	

Abbreviations: BF, breastfeeding; BMI, body mass index (calculated as weight in kilograms divided by height in meters squared); NA, not applicable.

^a^ The pre–Mama Sana group is the historical control.

^b^ Pre–Mama Sana group, n = 186; Mama Sana group, n = 123.

^c^ Pre–Mama Sana group, n = 211; Mama Sana group, n = 148.

### Breastfeeding Outcomes

**Figure 2.**
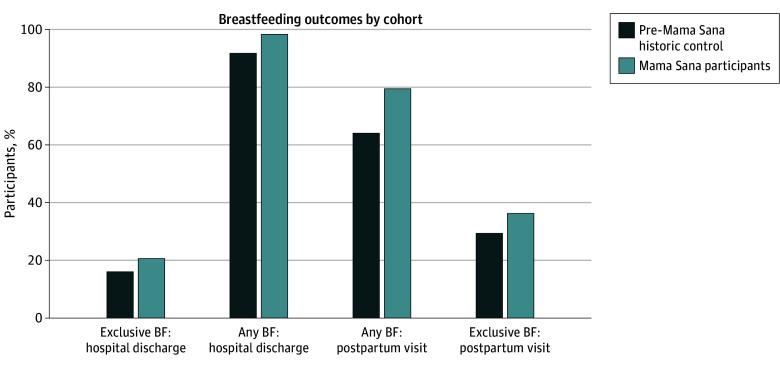
Breastfeeding (BF) Outcomes During the Delivery Hospitalization and Postpartum Visit (6 Weeks) Comparing Mama Sana Participants With a Historical Control

Compared with the historical control, Mama Sana participants had significantly higher absolute rates of any breastfeeding at hospital discharge (172 of 175 [98.3%] vs 222 of 242 [91.7%]; aRD, 6.6%; 95% CI, 2.6% to 10.5%), which was sustained at 6 weeks post partum (81 of 102 [79.4%] vs 109 of 170 [64.1%]); aRD, 15.3%; 95% CI, 4.6% to 26.0%) (Figure 2 and Table 2). In adjusted analysis, participation in Mama Sana was associated with higher rates of any breastfeeding at hospital discharge (RR, 1.07; 95% CI, 1.03 to 1.12; aRD 7.1%; 95% CI, 2.8% to 11.5%) and at 6 weeks post partum (RR, 1.24; 95% CI, 1.07 to 1.44; aRD, 15.6%; 95% CI, 4.8% to 26.4%) compared with historical control (Table 2). Rates of absolute EBF at hospital discharge (36 of 175 [20.6%] vs 39 of 242 [16.1%], RD (aRD, 4.5%; 95% CI, −3.1% to 12.0%) and at the 6-week postpartum visit (37 of 102 [36.3%] vs 50 of 170 [29.4%], RD (aRD, 6.9%; 95% CI, −4.7% to 18.4%) were higher for Mama Sana participants compared with historical control (Figure 2 and Table 2), but not significantly different. After adjustment, Mama Sana participation was not associated with EBF at hospital discharge or 6 weeks post partum (Table 2). There was no significant difference in BMI change.

**Table 2.** Breastfeeding Outcomes Comparing Mama Sana Participants to Pre–Mama Sana Group

Breastfeeding outcomes	Relative risk ratios (95% CI)		Risk difference, % (95% CI)	
	Unadjusted	Adjusted^a^	Unadjusted	Adjusted^a^
Exclusive breastfeeding at hospital discharge	1.28 (0.85 to 1.92)	1.34 (0.89 to 2.01)	4.5 (−3.1 to 12.0)	2.5 (−1.2 to 6.4)
Any breastfeeding at hospital discharge	1.07 (1.03 to 1.12)	1.07 (1.03 to 1.12)	6.6 (2.6 to 10.5)	7.1 (2.8 to 11.5)
Any breastfeeding at postpartum visit	1.24 (1.07 to 1.44)	1.03 (0.83 to 1.28)	15.3 (4.6 to 26.0)	15.6 (4.8 to 26.4)
Exclusive breastfeeding at postpartum visit	1.23 (0.87 to 1.74)	1.0 (0.7 to 1.5)	6.9 (−4.7 to 18.4)	6.2 (−5.4 to 17.8)

^a^ Adjusted for nulliparity, mode of delivery, breastfeeding within 1 hour.

### Process and Balancing Measures

More Mama Sana participants had lactation consults placed than the historical cohort (109 of 175 [62%] vs 108 of 242 [45%]; *P* < .001). All Mama Sana participants (175 of 175 [100%]) received breastfeeding counseling in Spanish (Table 3), compared with 49 of 242 (20%) in the historical control (*P* < .001). Of all patients in epoch 2, 74% received breastfeeding counseling in Spanish.

**Table 3.** Maternal Outcome, Process Measures, and Infant Measures Between Groups

Measures	Participants, No. (%)		*P* value
	Pre–Mama Sana, n = 242	Mama Sana (n = 175)	
Maternal BMI change at postpartum visit from prepregnancy BMI, mean (SD)^a^	1.1 (4.7)	0.9 (2.2)	.55
Process measures, No. (%)			
Lactation consult placed during delivery hospitalization	108 (44.6)	109 (62.3)	<.001
Lactation support during delivery hospitalization			
Any	124 (51.2)	175 (100)	<.001
Spanish (interpreter or Spanish-speaking)	49 (20.3)	175 (100)	<.001
Language-concordant	5 (2.1)	172 (98.3)	<.001
Infant measures			
Hyperbilirubinemia	21 (8.6)	7 (4.0)	.048
Hypoglycemia requiring nonformula treatment	11 (4.5)	4 (2.3)	.29
% Weight loss at discharge, mean (SD)	3.7 (2.7)	3.9 (3.1)	.51

Abbreviation: BMI, body mass index (calculated as weight in kilograms divided by height in meters squared).

^a^ Pre–Mama Sana group, n = 113; Mama Sana group, n = 83.

### Secondary Analyses

There was no difference in breastfeeding outcomes between epochs overall, either at hospital discharge or at 6 weeks post partum (eTable 2 in Supplement 1). Lactation consult placement and access to lactation counseling, both overall and Spanish language-specific, increased significantly from epoch 1 to epoch 2 (eTable 3 in Supplement 1). Additionally, there was no difference in hypoglycemia and weight loss at discharge between epochs. Fewer infants had hyperbilirubinemia in epoch 2.

## Discussion

Mama Sana was developed in response to stark disparities in breastfeeding rates at our academic tertiary level hospital. To our knowledge, this is one of the first evaluations of an inpatient hospital-based program to improve breastfeeding outcomes through language-concordant lactation counseling for Spanish-speaking Hispanic patients. Mama Sana was associated with higher rates of any breastfeeding at hospital discharge and 6 weeks post partum and more lactation support access for participants. However, there were not significantly higher rates of exclusive breastfeeding at hospital discharge. Despite this, Mama Sana has the potential to reduce health disparities throughout the life course and provide a model for other hospital initiatives targeted toward Spanish-speaking Hispanic populations.

Access to lactation support has been shown to improve breastfeeding outcomes for mothers from minoritized racial and ethnic groups.^17,32,33^ In a randomized clinical trial, Bonuck et al,^26^ found that providing prenatal and postnatal lactation consults (in-hospital and home visits) increased any breastfeeding through 5 months of life. Additional studies have shown that breastfeeding peer counseling can increase breastfeeding initiation^34^ and exclusivity^35^ in the first 3 months of life. Peer counselors share cultural experiences with the breastfeeding parent and can share personal suggestions about overcoming cultural and structural barriers to breastfeeding; these long-term relationships lead to increased breastfeeding duration and exclusivity.^32^ Mama Sana aimed to foster this type of relationship with a bilingual, Hispanic CLC, offering CLAS with substantial clinical expertise, novel compared with other lactation intervention studies among Hispanic patients. Rates of any breastfeeding during the delivery hospitalization and at 6 weeks post partum were higher among Mama Sana participants, potentially associated with improved in-hospital and postpartum lactation support, respectively. Our findings were therefore consistent with other studies demonstrating benefits of lactation support in increasing breastfeeding initiation and continuation.^26,34,35,36^ Distinctly, Mama Sana participants had higher in-hospital breastfeeding rates with hospital-based counseling without a prenatal component. EBF rates during the delivery hospitalization and at the 6-week postpartum visit trended higher for participants but were not statistically significant, highlighting the need for further program optimization.

Mama Sana’s current setting (primarily hospital-based at birth) allows for unique assessment of CLAS lactation education during the critical immediate postnatal period, with outpatient lactation support targeting breastfeeding continuation limited to postpartum telephone follow-up. Programs that have successfully increased breastfeeding duration and exclusivity have had more frequent and prolonged postnatal interaction with mothers,^36,37^ often at locations they might otherwise be visiting, such as pediatric clinics. Addressing the antenatal to postpartum care continuum is also crucial to impacting breastfeeding duration and exclusivity.^38,39^ The most effective visit timing and frequency has yet to be determined, but we hope that implementation of subsequent phases of Mama Sana, which include prenatal visits and robust longer term postpartum follow-up, provides insight.

There were some drivers of EBF rates that we were unable to assess or impact in this program, such as breastfeeding intention. Past work has shown that Hispanic mothers have significantly higher intention to breastfeed at all time points than other racial and ethnic groups but are less likely to meet those goals.^39^ However, in interviews, Latina mothers in Massachusetts described *las dos cosas* (both formula and breastfeeding). They introduced formula for many reasons, including low milk supply, perception of infants feeling fuller, and anticipated return to work, but did not understand the connection between formula and milk supply or breast milk’s dose-response health benefits.^40^ Culturally and linguistically concordant counseling has the potential to improve education about EBF benefits.^41^ Mama Sana aimed to mitigate the breastfeeding drop-off through increased availability of trained lactation counselors, which may improve quality and quantity of breastfeeding counseling for women from minoritized racial and ethnic groups in the US.^36,38,42^ However, improved antenatal counseling is needed, and introducing group breastfeeding classes may allow for important peer or community connections that can facilitate continued breastfeeding support. Additionally, programs need to be bolstered by policies, such as paid parental leave and time for pumping, to best support breastfeeding exclusivity and continuation.^43^

Breastfeeding decision-making is complex, and the decision and ability to exclusively breastfeed involve many environmental and social contributors. Social support from family and peers can contribute to breastfeeding continuation and exclusivity. Engaging family (especially fathers) and community-based support are important parts of evidence-based interventions to increase breastfeeding.^38^ Parental leave in the postpartum period can support increased breastfeeding duration.^44^ Transitioning back to work can also be specifically challenging. Several resources are required to support breastfeeding in the workplace,^45,46^ including space to pump, access to refrigeration for storing milk, and flexible remote work policies. These are other potential targets for future out-of-hospital interventions.

### Limitations

This study has limitations. First, this is an observational, ecologic analysis, and there may be population and temporal factors that impact breastfeeding rates. This is particularly true given the impact of the COVID-19 pandemic on the historical control. However, the comparison of characteristics between periods did not reveal significant differences, and we adjusted for a priori factors prognostic of successful breastfeeding in our multivariate models. We recognize there may be residual unassessed confounders. There may have been selection bias in program referrals, given the higher likelihood of primiparous participants. However, Mama Sana was a care bundle that included lactation support, linguistic concordance, and human connection, factors that cannot be fully quantified or disentangled along the causative pathway. Classification of donor milk as breast milk, per Joint Commission on Accreditation of Healthcare Organizations guidelines, may lead to miscategorization of exclusive breastfeeding. When assessing continued program impact, several participants did not have outcomes documented at their postpartum visit, and therefore, our analysis is underpowered to detect differences in these outcomes. Finally, due to data availability, our data excludes some factors affecting breastfeeding, such as breastfeeding intention, health-related social needs, and workplace factors. However, we did include a range of factors affecting breastfeeding in our adjusted models.

In recognition of the need for a lactation support continuum (prenatal discussion through post-discharge support), Mama Sana expanded its offerings to include prenatal breastfeeding group classes for interested patients. To address other contributors to dyadic health, culturally and linguistically tailored nutrition classes were made available prenatally. We also appreciate that the needs of a population served by an obstetric service in a tertiary care center may not be generalized to other health care settings, and hope to expand to other care settings.

## Conclusions

In this cohort study, the provision of language-concordant, culturally tailored lactation support through Mama Sana was associated with improved rates of any breastfeeding during the delivery hospitalization and at the postpartum visit, although the intervention did not improve the main outcome of EBF. Mama Sana also improved access to Spanish-language breastfeeding counseling, suggesting a path to equitable provision of perinatal care for Spanish-speaking Hispanic patients and reducing health inequities. This study is important in reducing breastfeeding disparities and improving lactation support at a high-risk obstetric center. Ultimately, the positive health effects of breastfeeding compound over the life course, and Mama Sana’s implementation has the potential to have downstream positive effects for the parent-infant dyad.

## Notes

Supplement 1. **eTable 1.** Baseline Characteristics of Spanish-Speaking Mother-Infant Dyads at Brigham and Women’s Hospital in Epoch 2 (January 2022 to September 2023), by Participation in Mama Sana **eTable 2.** Associations of Breastfeeding Outcomes Comparing All Patients After Implementation of Mama Sana (Epoch 2, January 2022 to September 2023, 319 dyads) to Historical Control Before Implementation of Mama Sana (Epoch 1, July 2019 to December 2021, 242 Dyads, Reference) **eTable 3.** Maternal Outcome, Process Measures, and Infant Measures by Epoch

Supplement 2.
Data Sharing Statement
