# Supplementary material for: Implementation of a Language-Concordant, Culturally Tailored Inpatient Lactation Program
Source: JAMA Netw Open. 2025 Mar 7;8(3):e250274. doi: 10.1001/jamanetworkopen.2025.0274 (PMC11889473; doi:10.1001/jamanetworkopen.2025.0274)
Supplement: Supplement 1. — eTable 1. Baseline Characteristics of Spanish-Speaking Mother-Infant Dyads at Brigham and Women’s Hospital in Epoch 2 (January 2022 to September 2023), by Participation in Mama Sana eTable 2. Associations of Breastfeeding Outcomes Comparing All Patients After Implementation of Mama Sana (Epoch 2, January 2022 to September 2023, 319 dyads) to Historical Control Before Implementation of Mama Sana (Epoch 1, July 2019 to December 2021, 242 Dyads, Reference) eTable 3. Maternal Outcome, Process Measures, and Infant Measures by Epoch [file jamanetwopen-e250274-s001.pdf]

Supplemental Online Content

Kalluri NS, Padilla-Garza E, Kehoe T, et al. Mama sana, “healthy mother”, a language-concordant, culturally tailored inpatient lactation program. *JAMA Netw Open*. 2025;8(3):e250274. doi:10.1001/jamanetworkopen.2025.0274

eTable 1. Baseline Characteristics of Spanish-Speaking Mother-Infant Dyads at Brigham and Women’s Hospital in Epoch 2 (January 2022 to September 2023), by Participation in Mama Sana
eTable 2. Associations of Breastfeeding Outcomes Comparing All Patients After Implementation of Mama Sana (Epoch 2, January 2022 to September 2023, 319 dyads) to Historical Control Before Implementation of Mama Sana (Epoch 1, July 2019 to December 2021, 242 Dyads, Reference)
eTable 3. Maternal Outcome, Process Measures, and Infant Measures by Epoch

This supplemental material has been provided by the authors to give readers additional information about their work.

© 2025 Kalluri NS et al. *JAMA Network Open*.

eTable 1. Baseline Characteristics of Spanish-Speaking Mother-Infant Dyads at Brigham and Women's Hospital in Epoch 2 (January 2022 to September 2023), by Participation in Mama Sana

**Maternal and Perinatal Characteristics** [patients (n, %)]	**Not seen by Mama Sana** *n = 144*	**Seen by Mama Sana** (Epoch 2a) *n = 175*	**p-value**
Age, mean (SD), y	29.4 (5.7)	28.9 (6.1)	0.48
Multiparous	105 (72.9)	98 (56.0)	0.002
C-section delivery	40 (27.8)	63 (36.0)	0.12
Diabetes during Pregnancy	15 (10.4)	11 (6.3)	0.18
Maternal Hypertension	7 (4.9)	9 (5.1)	0.91
Pre-Eclampsia	2 (1.4)	3 (1.7)	1.0
Pre-Pregnancy BMI, mean (SD)<br>n = 106; n = 123	27.4 (5.4)	27.6 (5.6)	0.72
Skin-to-skin after delivery<br>n = 143; n = 171	107 (74.8)	117 (68.4)	0.21
BF within 1 hour	71 (49.3)	83 (47.7)	0.78
**Infant Characteristics** [mean, SD]			
GA at delivery, weeks	39.1 (1.1)	39.4 (1.2)	0.03
Birth Weight, kg	3.3 (0.4)	3.4 (0.4)	0.65
Female sex, n (%)	70 (48.6)	78 (44.6)	0.47
Male sex, n (%)	74 (51.4)	97 (55.4)	

© 2025 Kalluri NS et al. *JAMA Network Open*.

eTable 2. Associations of Breastfeeding Outcomes Comparing All Patients After Implementation of Mama Sana (Epoch 2, January 2022 to September 2023, 319 dyads) to Historical Control Before Implementation of Mama Sana (Epoch 1, July 2019 to December 2021, 242 Dyads, Reference)

Breastfeeding Outcomes	Relative Risk Ratios (95% CI)		Risk Difference (95% CI)	
	Unadjusted	Adjusted*	Unadjusted	Adjusted*
Exclusive breastfeeding at hospital discharge	1.19 (0.82, 1.71)	1.26 (0.88, 1.81)	3.0 (-3.3, 9.3)	2.8 (-2.6, 8.2)
Any breastfeeding at hospital discharge	1.01 (0.97, 1.07)	1.02 (0.98, 1.07)	1.4 (-3.0, 5.8)	2.2 (-2.5, 6.9)
Any breastfeeding at postpartum visit	1.11 (0.97, 1.28)	1.12 (0.97, 1.28)	7.3 (-2.2, 16.8)	7.3 (-2.3, 16.9)
Exclusive breastfeeding at postpartum visit	1.12 (0.83, 1.52)	1.13 (0.83, 1.53)	3.6 (-5.8, 13.0)	2.8 (-6.7, 12.2)

*Adjusted for nulliparity, mode of delivery, and breastfeeding within 1 hour

© 2025 Kalluri NS et al. *JAMA Network Open*.

eTable 3. Maternal Outcome, Process Measures, and Infant Measures by Epoch

Measures	**Epoch 1** (pre-Mama Sana, 7/2019 - 12/2021)	**Epoch 2** (1/2022-9/2023)[1]	p-value
**Maternal Outcome** (mean, SD)	*n = 113*	*n = 163*	
BMI change at postpartum visit from pre-pregnancy BMI	1.1 (4.7)	1.3 (2.1)	0.72
**Process Measures** (n, %)	*n = 242*	*n = 319*	
Lactation Consult placed during delivery hospitalization	108 (44.6)	173 (54.2)	0.02
Lactation support during delivery hospitalization			
Any	124 (51.2)	265 (83.1)	<0.001
Spanish (interpreter or Spanish-speaking)	49 (20.2)	236 (73.9)	<0.001
Language-concordant	5 (2.0)	197 (61.7)	<0.001
**Infant Measures** (n, %)		*n = 318*	
Hyperbilirubinemia	21 (3.7)	14 (4.4)	0.04
Hypoglycemia requiring non-formula treatment	11 (4.5)	9 (2.8)	0.37
% weight loss at discharge (mean, SD)	3.7 (2.7)	4.0 (2.7)	0.21

[1]Epoch 2 includes all Spanish-speaking dyads after Mama Sana implementation, both those who received standard care as well as those who received Mama Sana lactation support

© 2025 Kalluri NS et al. *JAMA Network Open*.
