# Supplementary material for: Implementation of a Language-Concordant, Culturally Tailored Inpatient Lactation Program
Source: JAMA Netw Open. 2025 Mar 7;8(3):e250274. doi: 10.1001/jamanetworkopen.2025.0274 (PMC11889473; doi:10.1001/jamanetworkopen.2025.0274)
Supplement: Supplement 2. — Data Sharing Statement [file jamanetwopen-e250274-s002.pdf]

Data Sharing Statement

Kalluri. Implementation of a Language-Concordant, Culturally Tailored Inpatient Lactation Program. *JAMA Netw Open*. Published March 07, 2025.
doi:10.1001/jamanetworkopen.2025.0274

Data
Data available: No

Additional Information
Explanation for why data not available: Deidentified data available upon reasonable request
